# Finding Plastic Patches in Coastal Waters using Optical Satellite Data

**DOI:** 10.1038/s41598-020-62298-z

**Published:** 2020-04-23

**Authors:** Lauren Biermann, Daniel Clewley, Victor Martinez-Vicente, Konstantinos Topouzelis

**Affiliations:** 1 Plymouth Marine Laboratory, Prospect Place, Plymouth, UK; 2 Department of Marine Science, University of the Aegean, Mytilene, Greece

**Keywords:** Environmental sciences, Ocean sciences, Optics and photonics

## Abstract

Satellites collecting optical data offer a unique perspective from which to observe the problem of plastic litter in the marine environment, but few studies have successfully demonstrated their use for this purpose. For the first time, we show that patches of floating macroplastics are detectable in optical data acquired by the European Space Agency (ESA) Sentinel-2 satellites and, furthermore, are distinguishable from naturally occurring materials such as seaweed. We present case studies from four countries where suspected macroplastics were detected in Sentinel-2 Earth Observation data. Patches of materials on the ocean surface were highlighted using a novel Floating Debris Index (FDI) developed for the Sentinel-2 Multi-Spectral Instrument (MSI). In all cases, floating aggregations were detectable on sub-pixel scales, and appeared to be composed of a mix of seaweed, sea foam, and macroplastics. Building first steps toward a future monitoring system, we leveraged spectral shape to identify macroplastics, and a Naïve Bayes algorithm to classify mixed materials. Suspected plastics were successfully classified as plastics with an accuracy of 86%.

## Introduction

In a relatively short period of time, the attributes of plastic initially perceived to be positive characteristics - convenience and longevity - have shifted to pose a widespread environmental problem. Within the marine context, millions of tonnes of plastic enter our oceans annually as micro- to macroplastic litter^1–6^. The economic cost to marine natural capital alone is estimated to range from $3300–$33,000 per ton of plastic per year^7^.

Larger plastics entering ocean waters have two fates - floating on the surface, or sinking due to bio-fouling and/or ballasting^8,9^. If not removed by clean-up operations, macroplastics (>5 mm) may harm marine life through entanglement or ingestion, but will inevitably fragment and degrade into microplastics^10–13^. Being able to detect larger floating plastics in coastal waters before they become entangled, ingested, exported and/or fragmented, may help to answer key questions about sources, pathways and trends. Furthermore, within the context of an increasingly stressed marine environment, actions that highlight and reduce marine plastic pollution can be counted as investments toward the health and future resilience of our global marine ecosystem services^7^.

Research on plastic detection using airborne data^14,15^, models and theoretical studies^16^ have demonstrated the potential to detect macroplastics in optical data^17–22^. Satellite remote sensing is the leading technique for collecting high quality, standardised optical imagery on global scales. For detection of floating macroplastics in the marine environment, however, few studies have succeeded. Previously, limiting factors have included temporal, spatial and spectral coarseness of available data. For example, Landsat 8 provides 9 spectral bands at a spatial resolution of 30 m, with a temporal resolution of 16 days. Commercial satellites including SkySat and RapidEye collect imagery at sub-meter to 5 m spatial resolution, but this is across 3 to 5 spectral bands. With the launch of the Sentinel-2A and 2B Earth Observation satellites by the European Space Agency (ESA) in 2015 and 2017, respectively, resolution may have improved sufficiently for detection of floating macroplastics from low-earth orbit. The 12-band Multi-Spectral instrument (MSI) sensors aboard the Sentinel-2 satellites were primarily developed for terrestrial services. However, coverage includes global coastal waters at revisit times of 2 to 5 days. Furthermore, the high spatial resolution of up to 10 m allows for detection of ’small’ features and objects in the marine environment, including river plumes, boats, and patches (rafts) of macroalgae. We propose that patches of floating materials that include macroplastics can be added to this list. Additionally, that the spatial and spectral resolution of Sentinel-2 is sufficient for macroplastics to be distinguishable from natural sources of floating debris, and seawater itself.

In contrast with clear water, which is characteristically efficient at absorbing near infrared (NIR) to shortwave infrared (SWIR) light, floating materials including macroalgae and macroplastics reflect in the NIR^16,18,20,21,23,24^. Leveraging these spectral properties makes aggregated materials floating on the ocean surface visible from space. Topouzelis *et al*.^21^ recently demonstrated this with plastic targets deployed off Mytilene in Greece. Spectra measured by drone-borne cameras and the Sentinel-2 MSI confirmed that floating rafts composed of plastic bottles, bags, and fishing nets consistently reflected light in the NIR. Intensity of reflectance appeared to be primarily dependent on the proportion of floating plastic within pixels. Consequently, once water composes more than 50 to 70% of a given pixel, we see poor reflectance in the NIR^21^. In pixels filled with at least 30% of bottles or bags, or 50% of fishing net, the characteristic reflectance and absorption features of floating plastics are observable.

Individual pieces of marine litter will likely remain below detectable limits until a front, eddy, or other submesoscale feature entrains multiple items into a larger patch. In the ocean, natural and anthropogenic materials tend to be aggregated together; generating patches of mixed objects including natural sources of debris, and litter dominated by macroplastics^13,25–28^. Once aggregated into sufficiently large patches of varying shapes and sizes, detection from Sentinel-2 is possible.

The aim of this study was two-fold. First, to demonstrate that macroplastics are detectable in data collected by the Sentinel-2 satellites. Second, to classify macroplastics and natural materials likely to be aggregated within mixed patches of floating debris. Key to detecting floating materials on subpixel scales was an index we developed - the Floating Debris Index (FDI), and generation of spectral signatures to identify dominant materials within pixels. An automated probabilistic machine learning approach allowed for the classification of materials, demonstrating that macroplastics are distinguishable from natural sources of debris in relatively clear waters.

## Results

**Figure 1:**
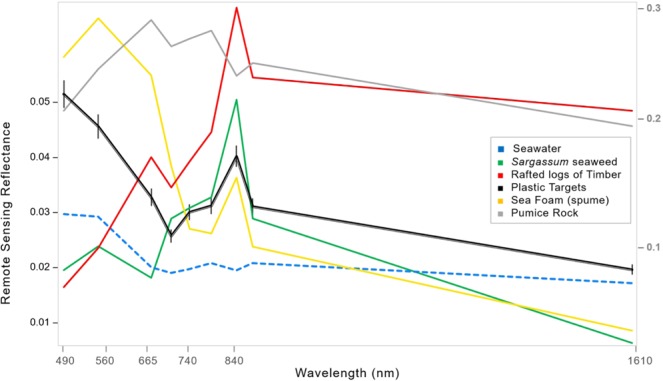
Spectral signatures derived from the mean spectra of deployed plastic targets (black line with error bars), seaweed representing floating vascular plants (green), seawater from all test sites (dashed blue line), rafted timber and wood representing non-photosynthetic plant materials (red), pumice representing non-plant debris (light grey), and spume representing sea foam, bubbles and froth (gold line). The x-axis shows the span of Sentinel-2 MSI bands from visible blue light at 490 nm, to short-wave infrared light at 1610 nm. The left-hand y-axis shows remote sensing reflectance (unitless) from Sentinel-2 for seawater, seaweed, sea foam and the plastic targets. Remote sensing reflectance (unitless) of timber and pumice was substantially higher. These were shifted lower to illustrate relative spectral shapes of all materials, and the corresponding reflectances are shown on the right hand y-axis in grey.

For remote sensing applications, spectral analysis refers to extraction of qualitative and quantitative information from the reflectance spectra of a given pixel, based on wavelength-dependent reflectance properties^29^. Classes of objects are therefore likely to have recognisable spectral features and characteristics, or spectral ’signatures’^30^. Based on absorption and reflectance patterns across 10 of the 12 Sentinel-2 MSI bands (from 490 nm to 1610 nm), we generated spectral signatures of detected seaweed, spume, timber, macroplastics and seawater. These proved key for identification of materials in mixed aggregations (Fig. 1).

As illustrated in Fig. 1, clear water is efficient at absorbing light in the near infrared (NIR). For the Sentinel-2 MSI this corresponds to a central wavelength at approximately 833 nm. Floating plastics and plants, on the other hand, both reflect at these wavelengths. The intensity of this reflectance signal in the NIR is dependent on how much each pixel is filled by material on subpixel scales^21^. Plastic shows a reflectance peak primarily in the NIR, while seaweed reflects light in the green (560 nm) and red edge (700–780 nm) bands too. Seaweed also appears to absorb SWIR light, relative to mean spectra of ocean water and plastic at 1610 nm, but variability here might be due to the atmospheric correction process. Timber shows a reflectance peak in the NIR, and also reflects relatively strongly in the red and SWIR. Pumice is noticeably bright across the optical range, reflecting in the red, red edge and SWIR, and absorbing in the NIR at approximately 833 nm. Finally, spume, which is likely to be composed of decomposing organic detritus (phytoplankton and algae, zooplankton, vascular plants), shows highest reflectance peaks in the green and red visible bands and a smaller peak in the NIR.

### Manual detection and identification of macroplastics in study sites using a novel floating debris index (FDI) and spectral signatures

Based on information of persistent or acute incidences of marine plastic litter in the scientific literature, popular press and social media, we selected the coastal waters off Ghana, North-West America, Vietnam, and the east coast of Scotland as case studies. Focus was on near-shore waters within these scenes, where aggregating features such as river plumes, fronts and/or eddies were visible in the red, green and blue (RGB) ‘true colour’ imagery. In all cases, though waters were not always clear or optically simple, they did not exhibit very high concentrations of Coloured dissolved organic matter (CDOM) or suspended sediment.

#### Accra, Ghana

The west African country Ghana boasts a coastline stretching about 550km, facing the Gulf of Guinea. Accra, the coastal capital, is home to around 3 million people and faces substantial waste management challenges, especially with regards to plastic litter^31^.

Applying the FDI to Sentinel-2 data acquired on the 31^st^ of October 2018, groups of bright pixels were detected along a front tracing the coastline. Materials were aggregated in floating patches that ranged from approximately 200 m to 6 km away from the coastline, and appeared to be dominated by macroalgae or spume. However, a proportion of pixels within detected aggregations conformed to the spectral signature of plastic (*n* = 23). These were identified as suspected ocean plastics.

#### Da Nang, Vietnam

In recent years, the coastal area off Da Nang has experienced a number of environmental challenges. Based on an internal monitoring report for Da Nang city coastal waters, action is being taken to minimise and prevent pollution from industrial and wastewater sources, and improve the environmental quality. Efforts from local and international institutions are also focused on tackling the problem of plastic pollution.

Application of the FDI algorithm highlighted bright pixels along both a river plume and frontal feature on the 30^th^ of October 2019. The majority of detected pixels tracing the river plume appeared to be composed of spume. A smaller proportion floating along the front and plume conformed to the spectral signature of plastic (*n* = 22), and these detections were identified as suspected ocean plastics.

#### Gulf Islands, Canada

Within waters of the Gulf Islands, large aggregations of floating debris tend to be primarily composed of woody material and macroalgae, as well as considerable quantities of foam, plastic bottles and bags, and cans^32^. In some cases, these mixed aggregations are so extensive, they are a known hazard to navigation in northern British Columbia and Alaskan waters.

Applying the FDI allowed for detection of floating debris south of Gabriola Island, which is part of the Gulf Islands in the Strait of Georgia in British Columbia (Canada). In an image collected on the 18^th^ of July 2018, bay-scale circulation appeared to entrain debris from the nearby marina, as well as woody material from timber rafting docks. Floating materials were aggregated in patches from around 180 m to 550 m away from Gabriola Island’s most southerly coastline, and high resolution imagery from Google Earth confirmed that floating aggregations recur here. A number of pixels within aggregations detected by the FDI conformed to the spectral signature of plastic (*n* = 26), and were identified as suspected ocean plastics.

#### Scotland, UK

A recent report on plastic pollution on UK beaches was circulated by the UK Marine Conservation Society, UK. Their findings showed that across 135 Scottish beaches, litter dominated by plastics and polystyrene had increased by 14% since 2017.

In a rare cloud-free and whitecap-free image acquired on the 20^th^ of April 2018, application of the FDI algorithm highlighted bright pixels along the edge of a strong front to the south-east of the Isle of May, and outside the rivers Tay and Esk. In both cases, floating aggregations were approximately 2 km–8 km away from the nearest coastline or island. Outside the rivers, the majority of pixels appeared to be composed of spume and seaweed. Particularly along the front, however, a number of pixels detected by the FDI conformed to the spectral signature of plastic (*n* = 23), and were identified as suspected ocean plastics.

### Automated classification of floating debris

Using the manually identified materials, an analysis was performed to develop a technique for automatically differentiating plastic from other materials within a Sentinel-2 image. The detected materials were analysed within a two-variable feature space by leveraging FDI values against another band ratio, the Normalised Difference Vegetation Index (NDVI). The combination of these two band ratios provide a simplified way to analyse data while keeping most of the information content of the 6 spectral bands of the MSI sensor.

**Figure 2:**
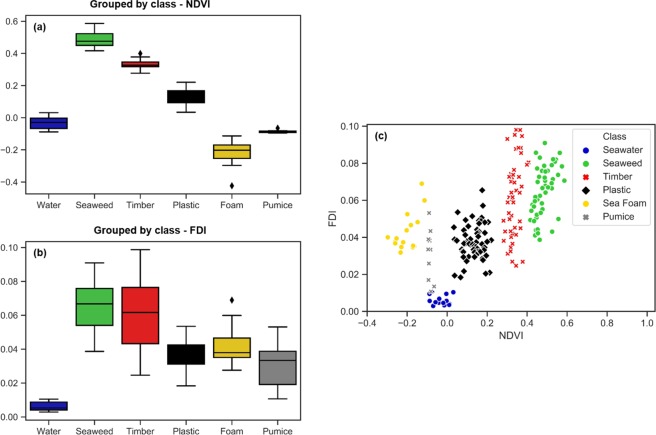
Classifying known floating materials in the marine environment. Using NDVI alone (**a**), we see that clear seawater (blue), wood (dark grey), spume (gold) and pumice (light grey) occupy distinct NDVI ranges that do not overlap with the combined (grouped) plastics. Unlike FDI (**b**), where values are primarily driven by how much of a given pixel is filled by material. Seaweed, timber wood, and foam detections show highest values here, suggesting that detected materials from these examples filled pixels completely, or to a high degree. Grouped plastics and pumice had some contribution from water and appeared to reply on subpixel detection. The plastic targets were detected on subpixel scales of 30–55%, as confirmed by drones. When visualised in 2-variable feature space (**c**) the combination of NDVI and FDI demonstrates distinct clustering of materials.

Plastic targets did not show a significant difference when examined with the validated plastics from Durban Harbour, particularly over the NDVI range (Kruskal-Wallis Test Chi square = 1.69, p = 0.19). Further, K-Means clustering partitioned both plastic targets and validated plastics into a single cluster. Thus, the two groups were combined for Fig. 2.

Using NDVI alone, the grouped plastics were distinguishable from seawater, seaweed, woody materials, sea foam and pumice (Fig. 2a). On the other hand, FDI values are primarily dependent on how much material composes a given pixel (Fig. 2b). When FDI and NDVI are examined together, however, materials show distinct clustering (Fig. 2c).

To provide an automated method for detection and classification of floating materials, a Naïve Bayes (Bayesian) algorithm was trained to predict plastics. The model was trained using the natural materials described and shown in Fig. 2, as well as the validated ocean plastics from Durban Harbour. The plastic targets deployed off Mytilene were not included in the training, and were instead used for validation of the Naïve Bayes classification algorithm.

**Figure 3:**
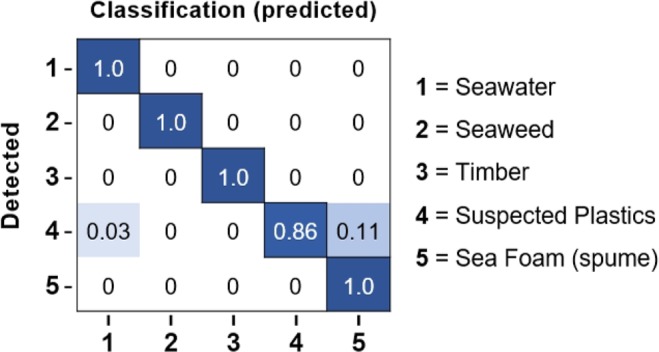
Normalised Confusion Matrix showing output from the Naïve Bayes classification test model. Suspected plastics (4) were predicted (classified) as plastics (4) by the model 86% of the time. Suspected plastics were classified as seawater (1) up to 3% of the time, or sea foam (5) 11% of the time. This suggests that some pixels were insufficiently filled with material when classified as water, or were dominated by spume or bubbles when classified as foam. The model did not classify any suspected plastics as timber or seaweed.

Applying the classifier to data not previously used for training (the detections from test cases) resulted in good agreement (Fig. 3), and the deployed targets from Greece were all correctly identified as plastic. Match-up between suspected plastics (i.e., manually identified as plastic but without *in situ* data to confirm) and those assigned as plastic by the classifier varied by approximately 20% between study sites. Detections that were suspected to be plastics but not identified as such by the model were instead classified as seawater, suggesting these pixels were not sufficiently filled by floating materials, or as spume. The full confusion matrix is shown in Fig. 3.

Across all sites, suspected plastics were classified as plastics by the Naïve Bayes model, with an accuracy of 86%. Detections off Accra and the Gulf Islands had highest agreement between suspected plastics and model classified plastics at 87% and 100%, respectively. Detections off Da Nang and Scotland showed agreements of 77% and 83%, respectively. For Accra, suspected plastics that were not classified as plastics were instead identified as seawater, suggesting that an insufficient amount of pixel was filled with floating debris. For both the Da Nang and Scotland case studies, a small proportion of suspected plastics were identified by the model as spume.

## Discussion

Based on the knowledge that the temporal, spectral and spatial resolution of ESA’s Sentinel-2 satellites is sufficient for observing small features in coastal waters, we tested two hypotheses. First, that floating macroplastics can be identified within mixed aggregations in the marine environment using a combination of our Floating Debris Index (FDI) and spectral signature. Second, that plastics can be automatically distinguished from natural sources of floating debris using a simple classification approach.

Our results strongly support these two hypotheses. First, application of our FDI for the Sentinel-2 Multi-Spectral Instrument (MSI) allowed for detection of materials floating on the ocean surface at sub-pixel scales. By applying the FDI to rafts composed of bags, bottles and nets deployed off Mytilene in Greece, we detected several useable pixels of plastic and generated a representative spectral signature. To build a library of spectral signatures, we then examined materials likely to be mixed with plastics in the marine environment, including seaweed, timber and sea foam. South of the island of Barbados, pixels filled with *Sargassum* seaweed were used to generate a spectral signature of floating plant material, and rafts of timber off British Columbia’s Gulf Islands were used to build a spectral signature of floating woody material. A spectral signature of foam or spume was generated outside of the Tay river mouth in Scotland and, opportunistically, rafts of pumice were used to make a spectral signature of floating non-plant materials.

Case studies where Sentinel-2 was used to detect aggregations likely to contain floating macroplastics included the east coast of Scotland (UK), Accra (Ghana), Durban (South Africa), Da Nang (Vietnam), and the Gulf Islands of British Columbia (Canada). In cases where our FDI highlighted aggregations of floating debris along fronts, river plumes or eddies, a number of pixels within patches closely resembled the spectral signature of plastic, and were identified as suspected plastics.

To then test the second hypothesis that marine macroplastics have sufficiently unique identifiers within the MSI spectral range, and can be distinguished from natural sources of debris, we used the Naïve Bayes classification algorithm. The highest uncertainty here stems from the limited number of scenes known to contain plastic, which can be used for analysis and model training. As far as we are aware, the Marine Remote Sensing Group from the University of the Aegean have provided some of the only *in situ* high-quality, standardised plastics data that can be used alongside Sentinel-2^21^ (Plastic Litter Projects 2018 and 2019). Increasing the number of known plastic targets across different water types would generate much-needed *in situ* validation of detections in Sentinel-2 imagery, and allow for better characterisation of the spectral signature of floating macroplastics. Additionally, deployment of larger targets composed of single-type and mixed plastic materials would circumvent reliance on subpixel detections, and aid future efforts to discern types of plastics detected in satellite data, respectively.

An unknown measure of uncertainty is also introduced at the atmospheric correction stage. The ACOLITE Dark Spectrum Fitting (DSF) algorithm was selected for this study after inter-comparison with POLYMER and Sen2Cor. Differences in output were evident, but without access to high quality *in-situ* measurements the significance of these differences remains uncertain. Work done by Topouzelis *et al*.^21^ demonstrates that both Sen2Cor and ACOLITE are suitable for the atmospheric correction, and for subsequent detection of macroplastics. For characterisation of floating materials in future studies, differences arising from atmospheric correction approaches may prove more important, especially in optically complex waters.

Despite the uncertainties, the results from this study support our methods for detecting, identifying and characterising macroplastic litter in coastal waters. We are the first to show reproducible success across four very different coastal areas, and our approach should be transferable to any remote sensing platforms with bands that are similar to those of Sentinel-2, including drones and future high-resolution satellite missions. While this work currently remains technically interesting - how optical remote sensors can be leveraged to find floating plastics - it is our hope that these results help to progress evidence-based decisions around the problem of plastics. Being able to detect marine litter close to land may aid clean-up operations before discarded items are exported, fragmented, or sunk below the surface of the water. From a model validation perspective, our results may also provide incremental steps toward answering unknowns around sources, pathways, fates, and trends.

Our next steps to improve on this early work are three-fold. First, we’re focusing on automating the manual steps for running plastics detection and classification across the Sentinel-2 data archive. Second, as the detection algorithms work most reliably in waters with relatively low turbidity, we recognise the need to tune detection methods for complex coastal and inland waters. Finally, to address uncertainties that arise from any adjacency effects and/or the atmospheric correction process, we aim to optimise or develop an approach that is best suited for plastics detection. Collecting *in situ* data from large rivers and highly tidal and turbid areas will be vital for these steps.

In terms of the Sentinel-2 sensors themselves, future improvements in the spectral signal to noise ratio, and access to high quality *in situ* data for validation would enhance the remote sensing of marine plastic litter significantly, and push forward automation on broadest geographical scales.

## Methods

### Sentinel-2 data access

Sentinel-2 is an Earth observation mission developed and operated by ESA under the Copernicus Programme. The Multi-Spectral Instruments (MSI) aboard Sentinel-2A and 2B work passively, and optical data is acquired along the orbital path at high spatial resolution (10 m, 20 m and 60 m) over land and adjoining coastal waters. Thought Copernicus program, MSI data are made available at no cost to users. We downloaded Level 1C products (at-sensor radiance) via the Copernicus and ESA Open Access Hub.

### Atmospheric correction

The inherent optical properties (IOPs) of floating materials can be leveraged for detection in Sentinel-2 imagery if NIR to SWIR wavelengths are conserved during the atmospheric correction process^16^. Ocean and atmospheric components (scattering and absorption) were subtracted from surface reflectance values using ACOLITE (Atmospheric Correction for OLI lite version 20181210.0). This marine atmospheric correction was developed for coastal waters using high resolution data from Landsat 8 and Sentinel-2, and the process is scene-based, requiring no previously defined ‘dark band’ like the NIR or SWIR. Instead, using the Dark Spectrum Fitting (DSF) algorithm, darkest pixels are dynamically selected based on multiple dark targets in a given image^33–35^. The DSF algorithm is described in detail in Vanhellemont and Ruddick^35^.

Output for surface reflectance (rhos, (*ρ*_*s*_) was computed using ACOLITE and visualised in the Sentinel Application Platform (SNAP) for further processing.

### Defining a floating debris index

At 10 m × 10 m, the highest spatial resolution of the Sentinel-2 Multi-Spectral Instrument, individual items of debris are likely to be below detectable limits until aggregated into patches. To enhance detection of patches floating on the ocean surface in Sentinel-2 imagery, we developed a floating debris index (FDI) using four of the twelve MSI bands (Table 1).

**Table 1:** Sentinel-2 MSI band characteristics, including descriptor, wavelengths and resolution.

MSI Band	Descriptor	S-2A Central Wavelength (nm)	S-2B Central Wavelength (nm)	Resolution (m)
Band 1	Coastal Aerosol	442.7	442.3	60
Band 2	Blue	492.4	492.1	10
Band 3	Green	559.8	559.0	10
**Band4**	**Red**	**664.6**	**665.0**	**10**
Band 5	Red Edge 1	704.1	703.8	20
**Band6**	**Red Edge2**	**740.5**	**739.1**	**20**
Band 7	Red Edge 3	782.8	779.7	20
**Band8**	**NIR**	**832.8**	**833.0**	**10**
Band 8a	Narrow NIR	864.7	864.0	20
Band 9	Water Vapour	945.1	943.2	60
Band 10	SWIR Cirrus	1373.5	1376.9	60
**Band11**	**SWIR1**	**1613.7**	**1610.4**	**20**
Band 12	SWIR 2	2202.4	2185.7	20

The selected bands for detecting floating debris are highlighted in bold.

The FDI was based on the Floating Algae Index (FAI) developed for Landsat, Medium Resolution Imaging Spectrometer (MERIS), and Moderate Resolution Imaging Spectroradiometer (MODIS)^18,36,37^. In place of the red band, where chlorophyll is most absorptive, we instead use the MSI Red Edge (RE) band at approximately 740 nm. Our debris detection index thus leverages the difference between the NIR, and the baseline reflectance of NIR. This baseline is derived from linear interpolation between the NIR-flanking RE2 and SWIR1 bands:

1 $$\begin{array}{lll}FDI & = & {R}_{rs,NIR}-{{R}^{{\prime} }}_{rs,NIR}\\ {{R}^{{\prime} }}_{rs,NIR} & = & {R}_{rs,RE2}+({R}_{rs,SWIR1}-{R}_{rs,RE2})\times \frac{({\lambda }_{NIR}-{\lambda }_{RED})}{({\lambda }_{SWIR1}-{\lambda }_{RED})}\times 10\end{array}$$

The subtraction of a baseline from the NIR reflectance serves to minimise sensitivity to changes in atmosphere and observation (aerosol type and thickness, solar/viewing angle, and glint), allowing for detection of floating objects through thin cloud or haze^36^.

The FDI was applied for subpixel detection of plastic targets deployed off Mytilene in Greece, as well as on dense floating patches of *Sargassum* seaweed off Barbados, rafted tree logs in waters off British Columbia, sea foam (spume) off the east coast of Scotland, and floating volcanic rock off Tonga. All materials were floating in relatively clear waters with low to moderate turbidity.

Simultaneously, we applied a Normalised Difference Vegetation Index (NDVI) to segregate floating vegetation from other materials:

2 $$NDVI=\frac{({R}_{rs,NIR}-{R}_{rs,RED})}{({R}_{rs,NIR}+{R}_{rs,RED})}$$

The NDVI is based on the fact that vegetation, including algae, show an increase in reflectance spectra at around 700 nm^36,38^. The difference between reflectance values in the NIR and red serves as a measure of photosynthetic capacity and/or density of vegetation. High NDVI values indicate dense patches of floating vegetation and/or high photosynthetic activity, while water generates low to negative NDVI values (no units).

### Study sites

To develop and test the method, Sentinel-2 scenes containing plastic were required. The Alfred Wegner Institute (AWI) LITTERBASE portal collates scientific studies that include or are focused on marine plastics, and summarises results in a mapped global format. We searched for studies that published data on floating macroplastics published after 2010. Social media sites Twitter and Instagram, were monitored for posts containing keywords pertaining to plastic pollution in riverine and marine environments. Keywords and hashtags included #marinelitter #marineplastic #plasticsoup #cleanup #plasticocean #plasticpollution #oceancleanup #cleanseas #keepouroceansclean #trashtag #plasticpollution.

**Figure 4:**
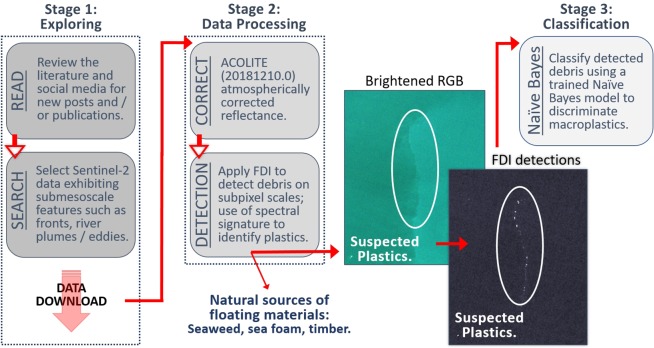
Flowchart illustrating and summarising steps required for detecting, identifying and classifying floating debris including macroplastics in Sentinel-2 imagery. Automation has been a key step, as this labour intensive process can take several hours from start to finish, per image. The Sentinel-2 satellite imagery in this figure show a front in the brightened RGB, and suspected plastics detected using the FDI. These are both from our Scotland case study from 20 April 2018. We generated our satellite imagery using the ESA open-source Sentinel Applications Platform (SNAP 6.0) software.

Based on the literature, scientific reports, news articles, and/or social media posts about marine litter posing an acute, increasing or persistent issue, several sites were identified (Table 2): the coastal waters off Accra (Ghana), the Gulf Islands of British Columbia (Canada), Da Nang (Vietnam), and the east coast of Scotland (United Kingdom) were examined for floating debris. A number of Sentinel-2 scenes were identified for each. Following the method shown in in Fig. 4, we focused on near-shore waters within these scenes, where aggregating features such as river plumes, fronts and/or eddies were visible in the ’true colour’ (RGB) imagery. Imagery exhibiting whitecaps were not used as these are also reflective in the solar spectral range^3,39,40^.

**Table 2:** Summary of the sites selected for training and testing, including how the information on each site was sourced, which submesoscale feature was present to group floating materials together (if applicable), and what the detected materials were, or were suspected to be.

Study Site	Training or Testing	Information Source	Aggregating Feature	Floating Materials
Accra, Ghana	Testing	Twitter	River plume, front	Suspected plastics
Barbados, Caribbean	Training	News and Twitter	N/A	Seaweed
Da Nang, Vietnam	Testing	Local Government	River plume, front	Suspected plastics
Durban, South Africa	Training	Instagram and personal communication	River plume	Validated plastics
Late Island, Tonga	Training	Twitter	N/A	Floating pumice rock
Mytilene, Greece	Testing and Training	University of Aegean	N/A	Deployed plastic targets
Gulf Islands, BC, Canada	Training	Eyes Over Puget Sound (EOPS) report	N/A	Rafted timber
Gulf Islands, BC, Canada	Testing	LITTERBASE portal, and scientific literature	Eddy	Suspected plastics
Scotland, UK	Training	Observations	River plume	Spume
Scotland, UK	Testing	Marine Conservation Society UK report	Front	Suspected plastics

### Determining spectral signatures of plastics and plants

During the Plastic Litter Projects (2018 and 2019), the Marine Remote Sensing Group from the University of the Aegean deployed floating targets of 5 × 5 m, 5 × 10 m, 10 × 10 m, and 5 × 20 m sizes. Targets were composed of plastics bags, bottles or fishing nets, and were deployed off Mytilene in Greece on the 7^th^ of June 2018^21^, 18^th^ of April, and 3^rd^ and 18^th^ of May 2019. Target detection in Sentinel-2 imagery acquired on these days was carried out at subpixel level using the FDI, which allowed for detection of 9 useable pixels in total. A spectral ‘signature’ for plastic was generated from the mean of these subpixel detections (*n* = 9).

To generate spectral identifiers for natural materials likely to be aggregated with floating macroplastics, rafts of *Sargassum* detected off Barbados on the 29^th^ of January 2019 were used to generate a mean spectral signature for seaweed. In many coastal waters, mixed aggregations are also known to include non-photosynthetic vegetation such as driftwood^41,42^. Thus, a mean spectral signature of timber or woody debris was generated from rafted logs floating in waters off British Columbia, Canada. Finally, foam (spume) from a river off the east coast of Scotland and aggregations of floating pumice off Tonga were used to generate mean spectral signatures of natural but non-plant floating materials (Fig. 1).

### Manual work flow

#### Supervised classification

After detecting suspected plastics using spectral signature, NDVI and the FDI, we tested if it was possible to discriminate different floating objects using a Naïve Bayes (Bayesian) classification. Although there are a number of supervised classification algorithms which could have been used, the Naïve Bayes algorithm was chosen as it requires only a small number of samples to train, and has demonstrated good performance compared to other algorithms^43^. This Naïve Bayes classifier is a probabilistic model, which relies on the assumption that predictors/features are independent - hence ‘naive’. For given values of FDI, NDVI and remote sensing reflectance, the classifier computes the probability of a detected pixel belonging to each of the classes, and assigns it to the one with the highest probability. We used the GaussianNB implementation within the Python scikit-learn library.

Features used for the classification were FDI and NDVI and remote sensing reflectance at 740 nm (Red Edge), 833 nm (NIR), and 1610 nm (SWIR). The remote sensing reflectance was included to aid differentiation of materials, rather than just using the band indices with the subset of wavelengths chosen as the ones showing the largest differences in the spectral shape.

The Naïve Bayes classifier was trained using: the validated plastics from Durban (*n* = 53), seaweed detections from around Barbados (*n* = 48), timber detections from British Columbia (*n* = 60), spume detections from Scotland (*n* = 17), and seawater from all of the above (*n* = 20). The plastic targets from Mytilene were not included in the training set, and were instead reserved for validation data in the testing dataset.

**Figure 5:**
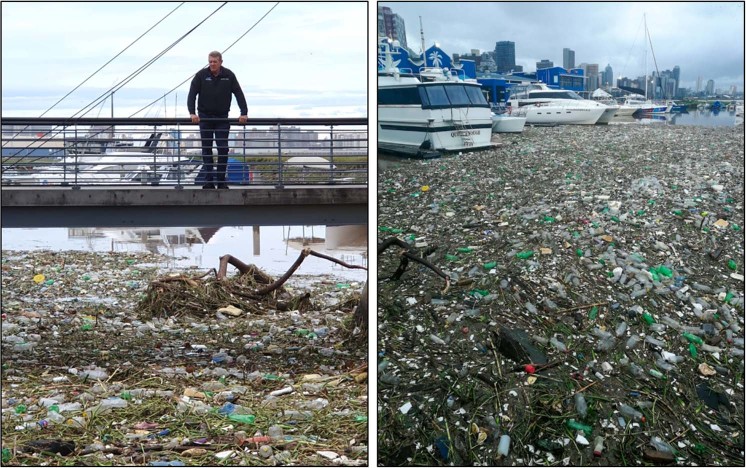
Substantial quantities of plastics and debris covered waters of Durban harbour on the 23^rd^ of April 2019, after flooding over the Easter weekend. Within two days, the macroplastics had been washed out to sea. Photos kindly shared with informed consent to publish.

The detections off Durban Harbour represented large quantities of plastic in a non-staged setting (i.e., plastics hadn’t been placed specifically for detection purposes). In this case, flooding had washed substantial quantities of plastic litter into the harbour. Photos posted to social media and reported in the news showed that the waters here were filled with floating macroplastics and plant debris on the 22^nd^ and 23^rd^ of April 2019 (Fig. 5).

Within two days, the floating plastics in particular had been washed into the sea and back along the beaches for “kilometres on end” (J Papendorf 2019, pers. comm., 21 June). Using the FDI, bright pixels were detected along fronts or plumes and through gaps in heavy cloud in a Sentinel-2B image acquired on the 24^th^ of April 2019. Over 50 pixels within the extensive floating debris patches matched the spectral signature of plastic. These were identified and classified as such. Using the 53 plastic detections from Durban to train the Naïve Bayes algorithm also allowed us to ensure (test) that the 9 known plastic targets were always identified as such during our classification process.
