# Author Correction: Finding Plastic Patches in Coastal Waters using Optical Satellite Data

**DOI:** 10.1038/s41598-020-64889-2

**Published:** 2020-05-12

**Authors:** Lauren Biermann, Daniel Clewley, Victor Martinez-Vicente, Konstantinos Topouzelis

**Affiliations:** 1 Plymouth Marine Laboratory, Prospect Place, Plymouth, UK; 2 Department of Marine Science, University of the Aegean, Mytilene, Greece

Correction to: *Scientific Reports* 10.1038/s41598-020-62298-z, published online 23 April 2020

The original version of this Article contained errors.

All instances of ‘San Juan Islands’ now read ‘Gulf Islands’.

Additionally, as a result of clerical error the wrong sign was used in the definition of R′_rs,NIR_ in Eq. (1), where the first instance of a minus should have been a plus:

1 $$\begin{array}{lll}FDI & = & {R}_{rs,NIR}-{{R}^{{\prime} }}_{rs,NIR}\\ {{R}^{{\prime} }}_{rs,NIR} & = & {R}_{rs,RE2}-({R}_{rs,SWIR1}-{R}_{rs,RE2})\times \frac{({\lambda }_{NIR}-{\lambda }_{RED})}{({\lambda }_{SWIR1}-{\lambda }_{RED})}\times 10\end{array}$$

now reads:

1 $$\begin{array}{lll}FDI & = & {R}_{rs,NIR}-{{R}^{{\prime} }}_{rs,NIR}\\ {{R}^{{\prime} }}_{rs,NIR} & = & {R}_{rs,RE2}+({R}_{rs,SWIR1}-{R}_{rs,RE2})\times \frac{({\lambda }_{NIR}-{\lambda }_{RED})}{({\lambda }_{SWIR1}-{\lambda }_{RED})}\times 10\end{array}$$

This has now been corrected in both PDF and HTML version of the Article.

